# Senses and repercussions of student assistance on the eating practices of students from a Brazilian public university during the COVID-19 pandemic

**DOI:** 10.3389/fpubh.2023.1168494

**Published:** 2023-06-19

**Authors:** Lara Vieira Constâncio Mota, Cláudia Roberta Bocca Santos, Flávia Milagres Campos

**Affiliations:** ^1^University restaurant, Fluminense Federal University, Niterói, Brazil; ^2^Postgraduate Program in Food and Nutritional Security, Nutrition School, Rio de Janeiro State Federal University, Rio de Janeiro, Brazil

**Keywords:** food access, higher education, social rights, public policy, dietary practices, social assistance, SARS-CoV-2, food and nutritional security

## Abstract

Student assistance (SA), regulated through the National Student Assistance Program (PNAES), seeks to meet the basic social needs of university students and is inserted in the field of public policies for higher education in federal institutions in Brazil. The program allocates financial resources in order to provide scholarships, housing, food, transport, physical and mental health, and accessibility for disabled students. The present study aims to identify the senses attributed by students of a federal public university to AE and the relationship between SA and their eating practices during the COVID-19 pandemic. A qualitative approach was used. Online questionnaire and focus groups were employed for data collection. The study public consisted of undergraduate students. Descriptive statistics and content analysis were used, opting for thematic analysis, with the support of the MAXQDA software. The core meanings were organized into two categories: (i) food during pandemic and (ii) role of student assistance. A total of 55 responses were obtained, and three focus groups were carried out. About 45% reported that the pecuniary aid offered by the university was the family’s only source of income during the pandemic and 65% used it to buy food. More than half described worsening in food quality, related to food prices. Although no specific evaluation instrument was used, it is plausible to admit that the students experienced a situation of food insecurity, given the uncertainty in regular access to food, the compromised quality of food, and the strategies mobilized to guarantee a minimum amount of food for all members of the family. Among the reported strategies were changing the location and mode of acquisition (receiving donations, buying in groups or in wholesalers) and choosing cheaper genres. Although students consider SA essential for access and maintenance at the university, a sense assigned to SA was the role of “help.” In general, students did not link SA to social rights, as part of public educational policy and as a mechanism for food and nutrition security. SA actions developed during the pandemic were essential for keeping students at the university, also functioning, albeit unintentionally, as a food and nutritional security mechanism.

## 1. Introduction

Student assistance (SA) aims to support students’ permanence in public education. In Brazil, the National Student Assistance Program (PNAES), under the authority of the Ministry of Education, guides actions in federal universities to minimize the effects of social and regional inequalities in the permanence and completion of higher education and raising graduation rates, through the adoption of complementary actions to promote improved academic performance, as well as reducing dropout rates and improving retention in higher education. According to Decree No. 7234 of July 19, 2010, which regulates the program, actions must be developed in the areas of housing, food, transportation, health care, digital inclusion, culture, sport, daycare, pedagogical support, participation and learning of students with disability, and global disorders of development and high abilities. The program aims to give priority to students from public schools and with a family income of up to 1.5 minimum wages *per capita*. For this purpose, the federal government allocates a specific resource each year to fund these actions at the federal universities (1).

In 2020, new challenges were imposed on universities by the corona virus (Sars-CoV-2) pandemic, with the temporary suspension of face-to-face classes and the functioning of university restaurants. In addition to financial aid, offering meals at low prices in university restaurants is one of the main actions related to food within the scope of PNAES and is widely adopted in higher education federal institutions in Brazil, as a form of indirect support (2). Since the university restaurants are considered public equipment for the promotion of food and nutritional security (3), their operation suspension, added to the change in the daily routine of academic activities, has an impact on the eating practices of students, especially those who depend directly on such equipment.

In facing this situation, besides maintaining the aid and scholarships in force before the pandemic, universities began to offer new aid on an emergency basis to meet the demands imposed by the measures necessary to contain the pandemic, with emphasis on support for emergency remote teaching activities (4–6).

Given this scenario, we seek to problematize important aspects of student assistance at the public university, facing the challenge of understanding how students perceive and use SA, what are their relations toward eating practices, and with the construction of the human right to adequate food, in a context of intense social and economic changes. The objective of this study was to identify the senses attributed by students of a Brazilian federal public university to student assistance actions and the relationship of these actions with their eating practices during the COVID-19 pandemic.

## 2. Methods

The study was carried out with students over 18 years of age, of both sexes, from undergraduate courses at the Fluminense Federal University (UFF). The university, located in the state of Rio de Janeiro, is headquartered in the city of Niterói and centers in 11 municipalities in the state. With about 63,000 students distributed in 129 graduation courses (7). The invitation to participate in the research was sent to all undergraduate students at UFF by email and made available on social networks (Instagram, Facebook) of student organizations.

A qualitative approach was used, aiming to understand meanings, values, and beliefs that in different ways interact with social systems and processes (8, 9). According to Mattos and Luz (10), the senses deal with the meaning, often non-rational, produced from an imaginary and symbolic universe, capable of producing identities and social relations. Thus, the senses express images and representations linked to actions experienced in everyday life.

The use of virtual means for data collection met the need for social distancing imposed by the pandemic. However, there was difficulty in scheduling data collection, which can be attributed to the ease of canceling or being absent from an appointment online. As instruments for data collection were used semi-structured online questionnaires and online focus groups (11). For the construction of the questionnaire, the Google Forms application was used. The questionnaire was divided into three blocks: (i) general information (age, gender, housing, and university admission), (ii) student assistance (inclusion in programs, meeting needs), and (iii) eating practices during the pandemic. The racial and ethnic information was self-report and, the classification used was based on a previous national survey (12), which included the categories: Asian descent, Black, Indigenous, Multiracial (descendant of the miscegenation between Black and White), and White. Regarding eating practices, it is considered that food is a historic-cultural creation of society (13); therefore, eating practices are composed of selection and way of acquiring food, place, and way of preparing and consuming meals.

In order to explore the data obtained through the questionnaire in-group interaction (11), focal groups were carried out, conducted by two moderators, who followed a previously elaborated script addressing experiences related to student assistance and students’ eating practices. The Google Meet video communication service was used to carry out the focus groups and with the participants permission; the sessions were recorded and later transcribed. The research was conducted with the consent of the Pro Dean of Student Affairs from UFF and was approved by the Research Ethics Committee of the Federal University of the State of Rio de Janeiro (CAAE: 46773021.1.0000.5285).

Descriptive statistics with variables expressed as a simple mean was employed in the analysis of objective questions of the questionnaire. For the discursive answers of the questionnaire and the transcripts of focus groups, the analysis was based on the framework of content analysis, opting through the use of thematic analysis (14). The encoding and processing of the corpus formed by the transcripts of the focus groups and questionnaire responses were carried out with the support of the MAXQDA software, version 2022.

The exploration of the material began with a floating reading and later, the definition of categories and classification of the constitutive elements of the corpus allowed the realization of inferences. The core meanings were recognized and organized into two categories: (i) Food during the COVID-19 pandemic and (ii) role of student assistance.

## 3. Results and discussion

### 3.1. Students and scholarships

It was obtained 55 responses to the questionnaire, and three focus groups were held with a total of 10 students, between August and September 2021. All who participated in the focus groups had previously responded to the quiz. Table 1 shows the participants’ data. Although the group of auto-declared White students alone is larger, Black and Multiracial students, when considered together, represent 54%. Most students accessed the university through what is known as quotas. According to Law No. 12.711, of August 29, 2012, at least 50% of federal university vacancies are reserved for public high school students, of which 50% must be from families with an income equal to or less than 1.5 minimum wages *per capita*. Also, a percentage is reserved for self-declared Black, Multiracial or Indigenous students, in a proportion at least equal to the presence of these groups in the total population of the Federation unit where the institution is located (15).

**Table 1:** Profile of the study participants.

Age (Years)	Responses (*n*: 50)^1^	Percentage (%)
18–28	37	74
29–39	9	18
40–50	2	4
51–60	2	4
Sex	Responses (*n*: 54)^1^	Percentage (%)
Feminine	42	77
Masculine	12	22
Race and ethnicity	Responses (*n*: 52)^1^	Percentage (%)
White	24	46
Black	9	17
Multiracial	19	37
Admission form	Responses (*n*: 45)^1^	Percentage (%)
With quota	28	62
Without quota	13	28
Others	4	8
Student scholarship	Responses (*n*: 49)^1^	Percentage (%)
Yes	33	67
No	18	30
Time student scholarship	Responses (*n*: 33) ^1^	Percentage (%)
Any less in 6 months	9	27
In 6–12 months	16	48
From 1–2 years	4	12
More in 2 years	4	12

1 Despite being 55 respondents, there was a variation in the number of responses for each question.

The identified profile is similar to that found in a national study on the socioeconomic and cultural profile of undergraduate students at Brazilian universities, carried out with 1,200,300 students, in which the percentage of admission through quota reached 50%, Black and Multiracial 51% and female 55% (12).

An expressive number of students belonged to the Nutrition course (21%), yet, if added, the participants belonging to the courses in the area of Social Sciences and Humanities represented the majority of respondents (56%). In addition, most participants were from the state of Rio de Janeiro (88%) and lived with their families (77%).

Although internal inequalities remain in federal universities, especially in the distribution of students among courses for more or less prestigious careers (16), it is possible to state that Brazilian higher education has become more accessible and diversified over the last few decades, as a consequence of public policies. In addition to affirmative actions, such as the vacancy reservation strategy since 2012, the Program for the Restructuring and Expansion of Federal Universities (REUNI) can be mentioned, which expanded the federal higher education network from 2007. Thus, the current characteristics of students is related to this movement toward the democratization of access to higher education in Brazil, based on the expansion of the number of vacancies, the opening of new campuses in the countryside, and the change in the selection criteria for incoming students. In this sense, the need to build an equity policy is highlighted, with attention to socioeconomic vulnerabilities and marginalization.

It is also observed in Table 1 that about two-thirds of the students received some scholarship from the SA, and the majority received less than 1 year. Table 2 lists the social programs run by UFF to attend students before the pandemic. It should be added that UFF regularly offers a psychological listening service to students.

**Table 2:** Social programs run by UFF before the pandemic.

Program	Characteristic
Transport assistance	Monthly payment
Food assistance	Financial resource for students whose *campuses* do not have university restaurant
Child care assistance	Monthly payment for students who have children
Housing assistance	Monthly payment
Food scholarship	50% reduction in the cost of meals in university restaurants
Permanence scholarship	Payment of financial aid directly to the student by the Federal Government
Emergency scholarship	Financial aid for up to 3 months
Academic development scholarship	Monthly payment
Athlete scholarship	Financial resources for the acquisition of material and participation in sports events
Support program for foreign students	Monthly payment
Support program for the student with deficiency	Financial resources for transportation expenses, acquisition of indispensable personal instruments, and support for studies.
Didactic material program	Grants access to the didactic materials
Reception assistance program	Monthly resource financial for incoming students

Elaborated based on data available on the institutional website of UFF.

In addition to these programs, which were generally maintained during the health crisis, there are those created to meet the demands of students with the start of the pandemic: (i) COVID-19 emergency aid, which grants monthly payment; (ii) Digital inclusion program, which is carried out through monthly financial aid for contracting a data package for internet access or through a concession/loan of devices and equipment (*chip,* modem); (iii) Chromebook loan, which is a temporary assignment of a laptop computer. The last two aimed to support remote emergency teaching activities, especially for students in social vulnerability, since the pandemic highlighted inequality in access to the internet, ownership of equipment, and mastery of information and communication technologies among students (5). The financial resources used by UFF to maintain the payment of scholarships and other aid to students during the pandemic were resources already provided for in the university’s budget or redirected for this purpose.

About 54% of the scholarship participants reported being benefited from the COVID-19 emergency aid offered by UFF, digital inclusion program, reception assistance program, and/or Chromebook loan. Part of the participants consider that aids and scholarships meet the demand for activities at the university (45%), and most report not having suffered from a suspension of payments of any assistance during the pandemic (86%).

### 3.2. Food and eating practices during the COVID-19 pandemic

The students’ eating practices, in general, were changed during the pandemic, and it was possible to identify the conditions that engendered such changes, as the strategies to guarantee food in adverse conditions.

**Figure 1:**
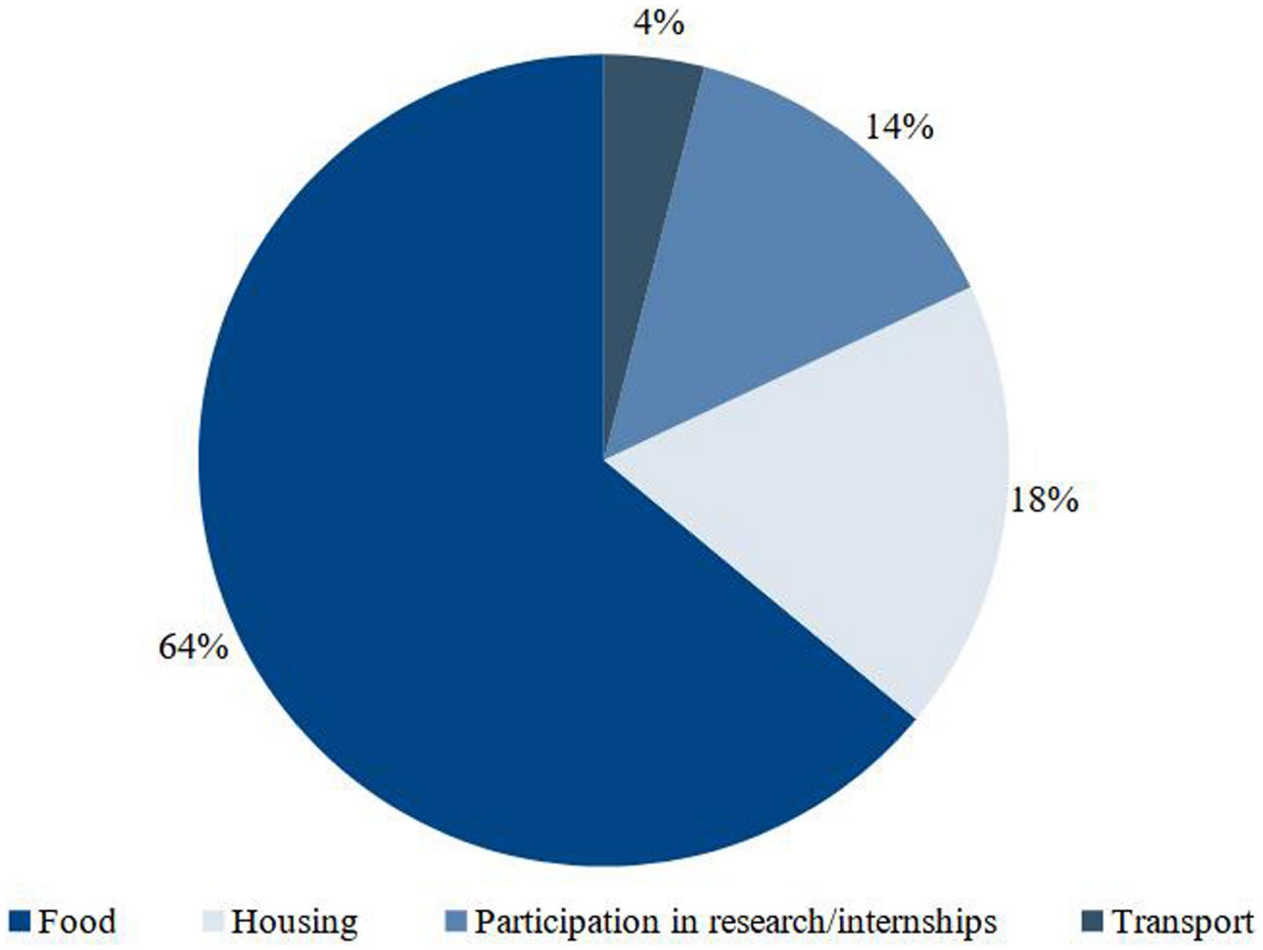
Destination of pecuniary amounts received from SA by students.

When asked if the assistance received from the university were the only source of income for the home/family during the pandemic, 45% answered yes, although it has not been studied how long this situation lasted. Among other sources of income, informal work (25%) and formal employment (22%) were reported. Scholarships and grants were intended for different needs, as shown in Figure 1. Scholarships and grants were intended for different needs, as shown in Figure 1. Among the 64% who reported using the financial resources received with food, 37% declared allocating 100% of the resources for this purpose. The majority (73%) reported that they could get the foodstuffs they wanted, but with restrictions due to high prices.

These results draw attention since participants faced unemployment in the family or obtained income from informal work, which puts them in a situation of vulnerability and risk of food and nutritional insecurity. The need for social isolation affected various activities in the productive sector, commerce and services, which not only increased unemployment rates, but also the activities of informal workers, who were already in a situation of lack of social security devices. The health crisis overlapped with socioeconomic inequalities in Brazil, with an increase in unemployment rates and food prices vs. a reduction in the population’s purchasing capacity (17, 18). It is known that this worsening of social inequality disproportionately affects individuals according to their social classes (19).

The data obtained through the questionnaire were corroborated by participation in the focus groups. With the pandemic, students began to live with a new reality, which ranged from the reduction in the purchasing capacity of food (due to lack of financial resources or difficult physical access), to routine modification, with the need for even closer daily coexistence with family members and/or partners.

“My grandmother started to live with us, and the food has become more expensive, my father was receiving less.”

“The period of isolation brought a little more financial difficulty. We moved house, and my family and I looked for sources of income until we were able to stabilize ourselves financially again.”

More than half of the participants (55%) reported a worsening in the quality of their diet during the pandemic, against 29% who reported an improvement. Among the factors that impacted eating practices were pointed out: the price of food (56%), pleasure in eating (16%), and lack of time (15%). Reports of changes in diet quality were also identified in the focus groups. The worsening was related to the disruption of routine and limited access to certain foods identified as healthier (fruits and vegetables), and such limitation was caused particularly by financial reasons such as unemployment, and stoppage in activities, in the case of professionals who worked as freelancers.

"I no longer eat meat, chicken, fish, eggs and sausages. I became vegetarian to save money."

“Here the quality of food has deteriorated, we used to eat more organically and now we buy more at supermarkets, but this has more to do with the financial issue.”

This deterioration in the quality of food was also found in other studies and was related to the increase in the consumption of processed foods and the purchase of fast food meals, linked to the so-called “harmful actions” such as the absence of physical activity, cigarette use, and alcoholic beverages (20, 21).

The notion that diet did not change or has improved during the pandemic is related to the possibility of having meals at home; having more time for food preparation, and reduction of certain foods identified as unhealthy (cookies, snacks, and fast foods).

“(…) I think I started to eat a little better (…). There were days when I didn’t even have lunch or dinner, I ate something on the street and at home I can always cook.”

The act of cooking and the improvement of culinary practices were reported in another study as a way to improve nutrition and reduce the anxiety generated by social isolation during the pandemic (22).

With the closure of several commercial establishments and the need for social isolation, 55% of the participants needed to change how and where they purchased food, and 56% changed the types of food purchased and preparations consumed. Receiving food in the form of donations, shares between neighbors, and use of vegetable gardens was declared by 42% of participants. Among the strategies created by students and their families to guarantee food in the face of the pandemic, it was possible to identify the replacement of certain foods considered more expensive (such as dairy, meat, and organic products) by cheaper ones, the purchase of food subdivided throughout the month to impact less on the budget, the purchase of staple food baskets instead of separated items, the elaboration of family or community vegetable gardens for sharing foods, the donation and division of staple food baskets between family members, and the replacement of the places of purchase of food, with the acquisition in wholesale networks.

"We stopped consuming some things, mainly some types of meat, yogurt, and reduced fruit intake."

“We started to buy on credit, and in smaller quantities in a marketplace next [house]. We took food in smaller amounts, and we pay at the end of the month."

Receiving staple food baskets and the sharing/exchange of food produced in gardens appears both as an element of public policy, when carried out by city halls, schools, and municipal reference centers of social assistance, and as an element of solidarity when part of the initiative of religious associations and friends.

"I have some friends who also have a vegetable garden then we exchange products. […] In that of you giving the other remembers you, it has been a period that people have remembered more that they are human beings, for at least here in mine street."

With the food crisis intensified by the pandemic, solidarity networks were arising as an option for survival, and guarantee of food, mainly in peripheral regions and communities. The creation of family and community gardens, the production of meals for distribution to all communities and neighborhoods, receiving and distributing of staple food baskets are some of the actions developed as an example of a food strategy and as a tool to reduce food insecurity (23).

Although no specific evaluation instrument was used, it is plausible to admit that the students experienced a situation of food and nutritional insecurity, given the uncertainty of regular access to food, the compromised quality of food, and the strategies mobilized to guarantee a minimum amount of food for all members of the family. These eating strategies were shown to be related to the drop in the income of the students’ families. A national survey carried out in Brazil in 2020 revealed that the loss of the job of a family member and family’s indebtedness are the two conditions that most impacted access to food during the pandemic, and 55% of the population coexisted with some degree of food insecurity (24). The same study showed that severe food insecurity was four times higher among those in informal work and six times higher among unemployed women.

A study carried out before the pandemic with 91 students at a Brazilian university found 94% of scholarship students at some level of food insecurity. The authors argue that food insecurity among university students needs to be recognized and enter the Brazilian public agenda (25).

The offer of meals in university restaurants, the main activity related to food developed by universities within the PNAES, was suspended due to the pandemic. However, the data show that, even indirectly, and in a limited way, the PNAES functioned as an important instrument in guaranteeing food for students during the pandemic. Many students used the financial resources received to guarantee food.

The PNAES does not seem to be an object of study of broad interest in the field of food and nutritional security research. In addition, it can be said that the program has a reductionist approach, lacking articulation with the food and nutrition security agenda. Considering that despite the attenuation of the health crisis after 2021, 59% of the Brazilian population still suffer from some degree of food insecurity, as revealed by a national survey carried out in 2021/2022 (26), the importance of food and nutritional security being the objective of public actions and policies is highlighted. We asked ourselves if regular access to food and the quality of food for students increased over the years 2020 and 2021 and how the PNAES can participate in a planned and permanent way in guaranteeing food and nutritional security for students.

Although it was not the initial purpose of the present study, in the scientific literature it is possible to identify works that recognized the relationship between the COVID-19 pandemic and mental health problems. Thus, a limitation of the analysis undertaken here was not addressing the relationship between food and mental health during the pandemic.

### 3.3. Student assistance: between aid and democratization of university access

Student assistance was identified by students as fundamental for the permanence of student socioeconomically vulnerable at university, especially during the pandemic of COVID-19. In general, the role of SA indicated by the students was centered on reducing the financial difficulties that impact the permanence and success of students, which is consistent with the focus of the PNAES.

The feelings related to SA, in general, were associated with gratitude and relief, reflecting the connotation given to it as “help essential and temporary for those in need” and an opportunity that does not benefit all. Reports of relief, tranquility, and security stand out, even if relative and temporary.

“[…] I believe that the role of student assistance is very strong even in person, (…) It is very important for us to maintain ourselves. The scholarship helps you to maybe even have a shorter period of work and be able to study more profitably.”

“The aid made me calmer, my concern started to be more with disciplines and less if it would have food at home."

The report of difficulties in accessing the SA was present in the questionnaire and in the focus groups, with the following being pointed out: the insufficient number of scholarships and other aids to meet the demand of students in social vulnerability, the insufficient value of the scholarships, the excessive demand for supporting documentation of lack, and complexity of procedures for requesting assistance. The changing trend in the profile of university students, characterized by the increase in people historically excluded from public higher education in Brazil—namely: Black and Multiracial people, graduates from public schools, and families with low income—puts pressure on the ability of the federal universities to meet the demand for SA, whether due to lack of resources or management difficulties. What ends up generating, according to Braga and Santos (27), the need for an “election of the poorest among the poor.” This perception, in some way, is also identified by the students themselves, as the excerpt illustrates:

“Despite having a focused character and having practically having to prove poverty, is a policy that brings us a perspective of a future. It's not great, but it saves lives.”

Although the limitations of the federal universities to deal with the demand of the SA are not only of a financial nature, one cannot neglect the context of the budget limitation destined for the education experimented in Brazil since 2017, which establishes spending restrictions and the freezing of expenses, impacting on the execution of the program of permanence in the federal universities (28, 29). In addition, the PNAES suffers direct threats of dismantling. In November 2021, the Education Ministry announced its intention to change the program with proposals for centralizing program monitoring and the use of meritocratic criteria for granting benefits, such as the exclusion of students who failed.

In the analysis of the reports of relief and gratitude regarding the SA, as well as the difficulties experienced to obtain the benefit, we refer to the notion of social suffering, from an anthropological perspective, as discussed by Victora (30). It turns, therefore, to social, political and institutional processes that produce suffering, from explicit or hidden conflicts and violence in everyday life. On the one hand, the public policy designed to serve vulnerable groups, as is the case of the PNAES, generates some relief from the suffering experienced by students, related to the uncertainty of what to eat, how to pay the bills, and how to stay in graduation. On the other hand, it intensifies the suffering in the selection of who should be a beneficiary, in the bureaucratic intricacies that students face to access the SA, and in the stigmatization that accompanies the benefit. Social suffering results from the oppression of the social structure itself and from the limitation of the subject’s ability to act. One cannot fail to recognize that the difficulty of access to food experienced by students represents a violation of a human right and a phenomenon that characterizes social suffering.

Students do not associate SA with the law, as part of the country’s educational policy and as a mechanism of food and nutritional security. We interpret gratitude as an effect of a welfarist conception, in which public apparatuses function to merely alleviate the difficulties of people in situations of social vulnerability, without participating in the process of reducing inequalities. SA, in this case, is characterized as a helper, a compensatory measure to mitigate social exclusion. If the meaning given to something has the capacity to produce identities, going beyond the conscious logic of the discourse, the welfarist meaning attributed by students to SA is attached to the image of the marginalized and of resignation. In this case, the emancipatory potential of SA is limited in terms of resistance to the forms of domination and exclusion present in the university, when it does not create possibilities for emancipatory struggles by the beneficiaries themselves.

However, yet in a less expressive way, part of the students recognize the relationship between assistance and permanence at the university, and understands its importance in the process of democratization of higher education.

"Many students if did not have access to assistance would not go to university. So I guess if there is no assistance, there is no student at the university and consequently there is no university. The public university has to be for everyone, if we don't give the student the opportunity to access public education then why do we have it?”

"Some students could not be in an online classroom since they were working at ifood for example.”

By punctuating the tension between studying or working during the pandemic, the student questions the failure not only of the SA but of the entire educational structure, which is unable to support the permanence in higher education of the student who works, without forgetting to mention that he works on their own, without any guarantee of social security.

There seems to be, therefore, a possibility for the construction and exercise of citizenship, in everyday life, from food, to recognizing the articulation of student assistance with the right to education and the human right to adequate food. The citizenship to which we refer is beyond the demarcations of societies organized into nation-states, under the command of a government that determines its policies (31). It is related to the feeling of belonging, political participation, the fulfillment of duties, and the fight for rights and for a dignified life, not only in the private sphere but also especially in public spaces. In this sense, identifying the articulation between SA and social rights such as education and food also allows recognizing that the violation of these rights disrespects the students’ own citizenship.

## 4. Conclusion

The profile of the study participants followed a national trend of change in the profile of university students in Brazil, with a higher percentage of Black and Multiracial students who entered the university through reservation of vacancies. In addition, most participants were aged between 18 and 28 years old, female and received some type of scholarship or aid from the SA.

With the pandemic, the university’s main activity related to food, which is offering meals at the university restaurant, had to be suspended. On the other hand, grants and emergency aid were offered. In general, students used these emergency grants to meet the demand for food.

More than half of the participants (55%) reported a worsening in the quality of their diet during the pandemic, especially related to high food prices, which led to the replacement of more expensive foods, considered of better quality, by cheaper ones, considered of lower quality. Changing the location and mode of purchase were the most reported strategies to ensure food for the whole family. It is worth noting the role of solidarity networks, especially in food sharing, in these strategies.

The identification of uncertainty in regular access to food, the impairment of its quality and the strategies mobilized to guarantee the family’s food allowed us to infer that the students were in a situation of food insecurity. By supplying part of the demand for food, in a way that was not properly planned, the PNAES ended up working to alleviate the food insecurity of these students. Despite this, it is understood that the isolated actions of the program are not able to cover all the needs of the contemplated students and, even more, they do not manage to reach the entire socioeconomically vulnerable group that accesses the university.

The students considered SA essential for access and permanence in the public university, especially during the pandemic. The senses about assistance were related to gratitude for the help received, not linking SA to social rights. On the other hand, more critical positions were identified regarding the need for greater investments in the PNAES and attention to factors that impact the lives of university students, such as the need for the double journey of studies and work.

The present research showed that, for the studied group, the PNAES assumed a central role for the survival of university students during the COVID-19 pandemic and not just for their permanence in undergraduate courses. However, the program does not seem to be a priority object of study in the scope of social policies, in particular, in Food and Nutritional Security Policies. It is hoped that the data presented here may contribute to legitimize the PNAES as an object of study and public policy that contributes to guaranteeing the human right to adequate food.

It should be noted that the PNAES is included in the public policy agenda for education and articulates with food and nutritional security policies. Think it from this approach can be fruitful, in the fight for its strengthening and qualification. At the same time, recognize this articulation between SA and the human right to adequate food could be a tangible possibility for the construction and the exercise of the citizenship at universities.

## Data availability statement

The raw data supporting the conclusions of this article will be made available by the authors, without undue reservation.

## Ethics statement

The studies involving human participants were reviewed and approved by Research Ethics Committee of the Federal University of the State of Rio de Janeiro Protocol: CAAE 46773021.1.0000.5285. The patients/participants provided their written informed consent to participate in this study.

## Author contributions

LM, CS, and FC: study conception and design, data analysis and interpretation, drafting, critical revision, and final approval of the manuscript. All authors contributed to the article and approved the submitted version.

## Conflict of interest

The authors declare that the research was conducted in the absence of any commercial or financial relationships that could be construed as a potential conflict of interest.

## Publisher’s note

All claims expressed in this article are solely those of the authors and do not necessarily represent those of their affiliated organizations, or those of the publisher, the editors and the reviewers. Any product that may be evaluated in this article, or claim that may be made by its manufacturer, is not guaranteed or endorsed by the publisher.
